# Colonic Duplication Cyst in an Adult Woman: A Case Report

**DOI:** 10.31729/jnma.5114

**Published:** 2020-11-30

**Authors:** Sundar Shrestha, Subodh Kumar Adhikari

**Affiliations:** 1 Department of General Surgery, Bir Hospital, Kathmandu, Nepal; 2 Department of Surgical Gastrointerology, Bir Hospital, NAMS, Kathmandu, Nepal

**Keywords:** *colonic duplication*, *colonoscopy*, *duplication cyst*, *hemicolectomy*

## Abstract

Intestinal duplications are rare congenital anomaly found in the pediatric age group. Although, the ileum is the most common site, there are cases of colonic duplications even in the adult. Colonoscopy is a good investigation tool for the diagnosis of colonic duplications; however, it may not be true in all cases. We report a case of 43 years woman presented with chronic constipation, intermittent colicky abdomen pain, and a cystic lump in the left abdomen diagnosed as tubular duplication cyst of descending colon. She was managed with left hemicolectomy and excision of the cyst with uneventful postoperative days. This case has been reported as it is a rare condition.

## INTRODUCTION

Gastrointestinal duplication cysts are rare congenital malformations generally found in less than two years of age. Ileum is the most common site (60%).^1^ They are rare in the colon (13%), only a few have been described in adults.^1^ They may go unrecognized until adulthood when it presents with chronic abdominal pain and constipation.^2^ They are of two types, cystic duplications (80%) and tubular duplications (20%).^1^ Complication ssuch as bleeding, melena, intussusception, obstruction perforation, or malignant degenerations are reported.^3^ The most common symptoms are mild abdominal pain with or without intestinal obstruction due to the direct compression of the adjacent bowel or distension of the duplication.

## CASE REPORT

A 43 years woman presented to gastro-surgery OPD in Blue Cross Hospital with painful cystic swelling on the left upper and lumbar region extending to the central and right upper abdomen associated with chronic constipation for 2 years. The pain was colicky, intermittent, mild, which was increasing in the recent months associated with a gradual increase in the size of the swelling. On examination, there was 12 cm x 8 cm, cystic swelling in the left hypochondrium, and the lumbar region extending up to the umbilical and right lumbar regions.

**Figure 1:**
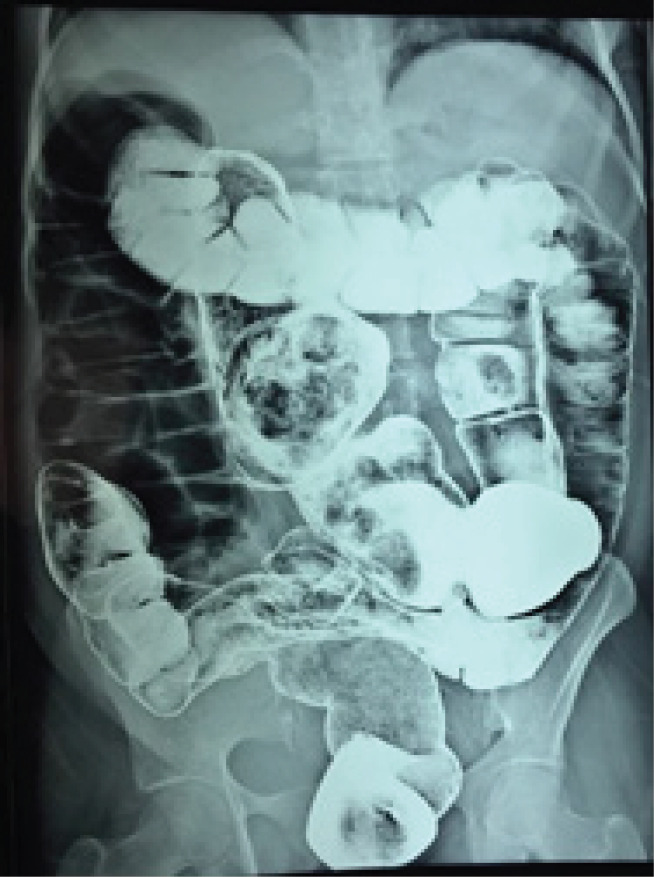
Barium enema showing a tubular duplication cyst of descending colon with a blind distal pouch attached to the retro-peritoneum.

Routine hematological and biochemical parameters were within normal limits. However, the ultrasonogram showed a 12 cm x 8 cm cystic lesion in the left lumbar region. Contrast-enhanced computed tomography (CECT) scan showed, large cystic dilation of large bowel involving transverse colon with gas and faces indicating a possible diagnosis of mega-colon. Colonoscopy showed normal findings. Barium enema revealed, duplication cyst arising from the distal transverse colon with the blind end extending up to the right upper quadrant (Figure 1).

**Figure 2:**
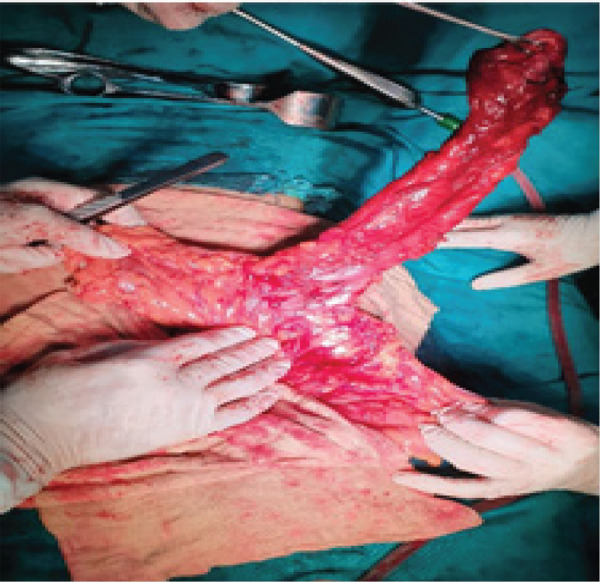
Intra-operative pictures showing duplication cyst with small communication ostium with a normal colon.

**Figure 3:**
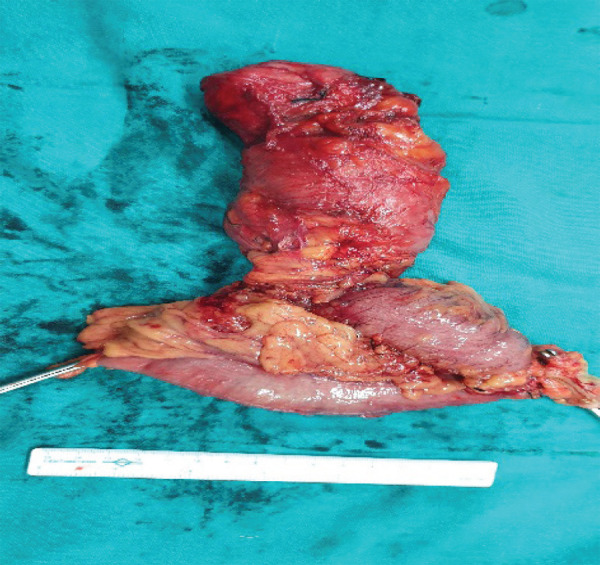
Resected specimen normal colon and tubular duplication cyst.

With the clinical diagnosis of the duplication cyst of the colon, she underwent left hemicolectomy and excision of the duplication cyst. Intraoperative findings revealed 35cm of tubular duplication cyst of the mid-transverse colon and the descending colon with the distal blind end attached to the retroperitoneum behind the second part of the duodenum (Figure 2 and 3).

The cyst and the normal functioning colon shared common vascular supply. There was a small communicating ostium between the main bowel and the cyst at the mid transverse colon.

**Figure 4:**
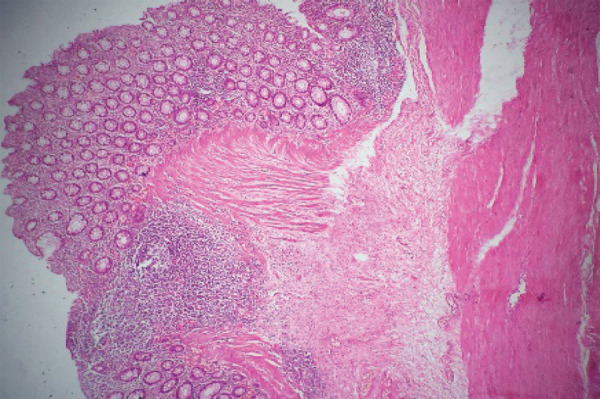
Microscopic pictures from the saccular part of the duplication cyst showing normal colonic mucosa, submucosa and thickened irregular muscularis propia.

The final pathological diagnosis was a tubular duplication cyst of descending colon with atrophic mucosa and few areas of strictures. Sections from the saccular duplicated colon show colonic tissue composed of mucosa, submucosa, muscularis propia, and serosa without any dysplasia or metaplasia. Muscularis propia is irregular and thickened (Figure 4).

The postoperative days were uneventful. The patient started on a liquid diet on the third postoperative day and was discharged on the sixth postoperative day. The patient was followed up after two weeks and 2 months on OPD, which were unremarkable.

## DISCUSSION

Enteric duplication cysts are rare congenital abnormalities commonly seen in pediatric populations, which occurs commonly in the small bowel. It has a smooth muscle coat and a mucosal lining.^4^ It is in the cystic form in more than 80% of cases and tubular in the rest. Colorectal duplication represents only 13% of all gastrointestinal duplications.^5^ Tubular duplication cysts are commonly associated with genitourinary abnormalities. There has been found an association of colonic duplication and colo-vesical fistula.^6^

It presents with a wide variety of clinical signs and symptoms causing difficulty in clinical and radiological diagnosis. The common presentation is colicky abdominal pain with or without abdominal distension or a lump and long-standing constipation. Now a day, the liberal use of newer diagnostic modalities like CT scan, MRI, and colonoscopy have helped to come to the diagnosis. Colonoscopy has reported being helpful in the case of larger ostium between the normal bowel and cyst.^7^ However, in our case colonoscopy has failed to diagnose the pathology possibly due to small communication between the cyst and normal colon.

Duplication cysts, if not associated with other abnormalities, may go undiagnosed until complication develops in adult life. It may be commonly mistaken for giant diverticulum, as its blind end appears large air-filled cysts as in our case. According to McNutt et al, the diverticulum is divided into three types:^8^ Type I: pseudo diverticulum with a wall consisting mainly of granulation tissue. Type II: inflammatory diverticulum, secondary to a perforation of the mucosa and submucosa communicating with the lumen. Type III: true diverticulum, all bowel layers, and communicating with the main lumen. Type III diverticulum is similar to a communicating duplication cyst. According to Choong and Frizelle, Type I diverticulum is a pseudo diverticulum with a fibrous wall with no muscular layer and type II is a true diverticulum with a muscular wall.^9^ Regardless of the classifications, giant colonic diverticulum may be difficult to distinguish from duplication cyst, when it becomes elongated. Our case was a tubular duplication cyst of the colon, as it was very elongated, not associated with other diverticular diseases of the colon, and lined by normal colonic mucosa free from dysplasia and metaplasia.

Recommended treatment for duplication cyst is resection of the part and anastomosis between the normal bowel either laparoscopic or open as it eliminates the risk of future malignancies in it. In our case, we performed left hemicolectomy due to the predominant part of the blood supply to the normal colon was from mesentery supplying to the cyst.

Our aim of reporting this case is to highlight the rarity of the condition, vague clinical presentations, and its difficulty in diagnosis with other entities. Clinical suspicion is necessary for an adult presenting with cystic abdominal lump with chronic constipation and colicky abdominal pain.

In a patient with chronic constipation, colicky abdominal pain, and the cystic lump in the abdomen, duplication cyst of the colon should be considered in the absence of other diverticular diseases as it presents with a wide spectrum of clinical signs and symptoms.
